# Comparing Twitter and Online Panels for Survey Recruitment of E-Cigarette Users and Smokers

**DOI:** 10.2196/jmir.6326

**Published:** 2016-11-15

**Authors:** Jamie Guillory, Annice Kim, Joe Murphy, Brian Bradfield, James Nonnemaker, Yuli Hsieh

**Affiliations:** ^1^ RTI International Research Triangle Park, NC United States

**Keywords:** social media, electronic cigarettes, tobacco, Twitter

## Abstract

**Background:**

E-cigarettes have rapidly increased in popularity in recent years, driven, at least in part, by marketing and word-of-mouth discussion on Twitter. Given the rapid proliferation of e-cigarettes, researchers need timely quantitative data from e-cigarette users and smokers who may see e-cigarettes as a cessation tool. Twitter provides an ideal platform for recruiting e-cigarette users and smokers who use Twitter. Online panels offer a second method of accessing this population, but they have been criticized for recruiting too few young adults, among whom e-cigarette use rates are highest.

**Objective:**

This study compares effectiveness of recruiting Twitter users who are e-cigarette users and smokers who have never used e-cigarettes via Twitter to online panelists provided by Qualtrics and explores how users recruited differ by demographics, e-cigarette use, and social media use.

**Methods:**

Participants were adults who had ever used e-cigarettes (n=278; male: 57.6%, 160/278; age: mean 34.26, SD 14.16 years) and smokers (n=102; male: 38.2%, 39/102; age: mean 42.80, SD 14.16 years) with public Twitter profiles. Participants were recruited via online panel (n=190) or promoted tweets using keyword targeting for e-cigarette users (n=190). Predictor variables were demographics (age, gender, education, race/ethnicity), e-cigarette use (eg, past 30-day e-cigarette use, e-cigarette puffs per day), social media use behaviors (eg, Twitter use frequency), and days to final survey completion from survey launch for Twitter versus panel. Recruitment method (Twitter, panel) was the dependent variable.

**Results:**

Across the total sample, participants were recruited more quickly via Twitter (incidence rate ratio=1.30, *P*=.02) than panel. Compared with young adult e-cigarette users (age 18-24 years), e-cigarette users aged 25 to 34 years (OR 0.01, 95% CI 0.00-0.60, *P*=.03) and 35 to 44 years (OR 0.01, 95% CI 0.00-0.51, *P*=.02) were more likely to be recruited via Twitter than panel. Smokers aged 35 to 44 years were less likely than those aged 18 to 24 years to be recruited via Twitter than panel (35-44: OR 0.03, 95% CI 0.00-0.49, *P*=.01). E-cigarette users who reported a greater number of e-cigarette puffs per day were more likely to be recruited via Twitter than panel compared to those who reported fewer puffs per day (OR 1.12, 95% CI 1.05-1.20, *P*=.001). With each one-unit increase in Twitter usage, e-cigarette users were 9.55 times (95% CI 2.28-40.00, *P*=.002) and smokers were 4.91 times (95% CI 1.90-12.74, *P*=.001) as likely to be recruited via Twitter than panel.

**Conclusions:**

Twitter ads were more time efficient than an online panel in recruiting e-cigarette users and smokers. In addition, Twitter provided access to younger adults, who were heavier users of e-cigarettes and Twitter. Recruiting via social media and online panel in combination offered access to a more diverse population of participants.

## Introduction

E-cigarettes have rapidly increased in popularity in recent years. In the United States, 13% of adults are e-cigarette users [1], marking a more than sevenfold increase from 2010 adult use rates [2]. Awareness of e-cigarettes is also widespread and has more than doubled among US adults since 2008 [3]. Many e-cigarette users believe that e-cigarettes will help with cessation [4-6], although population-based studies suggest otherwise. These studies demonstrate lower levels of cessation among smokers who use e-cigarettes compared with smokers who do not use e-cigarettes [3,7-10].

The proliferation of e-cigarettes in the United States has been driven, at least in part, by marketing and word-of-mouth discussion on Twitter [11]. Twitter content about e-cigarettes is primarily from marketers and advertisers, with two recent studies finding that at least 90% of tweets related to e-cigarettes were marketing or advertising tweets [11,12]. Although marketing and advertising expenditures for e-cigarettes have increased substantially across traditional and new media channels (television, magazines, out of home, radio, digital media)—256% between 2011 and 2013—people are most likely to hear about e-cigarettes online (41%) or from someone they know (35%) [13].

Twitter provides a free and efficient means of sharing and accessing information about e-cigarettes, making it a unique and informative vantage point from which to understand how people are using, selling, buying, accessing, and sharing information about these emerging products. At the same time, a growing body of literature demonstrates that social media provides an efficient and cost-effective space for recruiting hard-to-reach populations for survey research [14-20].

Social media sites, such as Facebook, Instagram, and Twitter, offer powerful targeting capabilities that aid in recruiting hard-to-reach populations [21,22], such as e-cigarette users and smokers, who make up 13% and 17% of the US adult population, respectively [1,23]. Targeting tools on social media allow researchers to target advertisements to users with specific demographic characteristics (eg, age, gender, income, education) and interests (eg, e-cigarettes, photography, smoking, folk music) based on their behaviors on these sites (eg, tweets, likes, comments, shares, retweets), and other third-party sites that they connect to through their social media accounts (eg, sharing an article from a news outlet’s website on Twitter, logging into an e-commerce website via Facebook). These capabilities reduce the time and resources required to recruit hard-to-reach populations for participation in research.

The majority of published studies that explore the use of social media for participant recruitment have used Facebook [14,16-20]. Facebook is a powerful tool for recruiting participants, but privacy restrictions, which are a default setting for all Facebook profiles, prevent researchers from gaining access to information that people share and are exposed to on Facebook. On Twitter, such information can be accessed if the Twitter user has a public profile (more than 90% of Twitter users have public profiles [24]) and consents to share his or her public Twitter data. Studies have analyzed public Twitter users’ data related to e-cigarettes [11,12], but no study to date has combined Twitter users’ data related to e-cigarettes (eg, tweets, followers, Twitter handles they follow) with self-reported survey data from these users. Combining these data sources would provide a more holistic understanding of how information about e-cigarettes is disseminated to e-cigarette users and smokers on Twitter; how these individuals differ based on demographic characteristics, e-cigarette use, and social media use; and how this information may influence perceptions and behavior related to e-cigarettes.

Given the rapid proliferation of e-cigarettes, researchers need timely data from e-cigarette users and smokers who have never used e-cigarettes, but may seek out e-cigarettes for cessation. Twitter provides an efficient and effective recruitment method because it allows for access to Twitter users’ public Twitter data and survey data and because it is a space where many conversations about e-cigarettes are occurring, both marketing and organic word-of-mouth conversations [11,12]. Although these features make Twitter a particularly appealing tool for participant recruitment, few published studies have used Twitter for participant recruitment [25]. Online panels offer a second method of accessing hard-to-reach populations because they have access to additional data from panel members (eg, age, smoking status, social media use) based on their responses to previous surveys. Although online panels have been criticized for recruiting too few young adults [26], among whom e-cigarette use rates are highest, they may provide a useful supplement to Twitter for recruiting smokers and e-cigarette users.

Twitter is more popular among people who are younger [27], thus we expect that younger adults will be more likely to be recruited for survey research via Twitter than online panel compared to adults older than 24 years. In addition, because e-cigarette use rates are highest among young adults [28], we expect that a larger number of e-cigarette users to be recruited via Twitter than online panel. Similarly, online panels tend to recruit people who are older on average [22] and smoking rates are highest among adults aged between 25 and 44 years [23]; thus, we expect a larger number of smokers to be recruited via online panel than Twitter. This study compares the efficacy and time efficiency of recruiting e-cigarette users and smokers via Twitter to online panel and explores how users recruited via these two methods differ by demographics, e-cigarette use, and social media use.

## Methods

### Participants

Eligible participants were adults who reported having ever used e-cigarettes (n=278; male: 57.6%, 160/278; age: mean 34.26, SD 14.16 years) and adult current smokers who reported current smoking every day or some days, having smoked ≥100 cigarettes in their lifetime, and having never used e-cigarettes (n=102; male: 38.2%, 39/102; age: mean 42.80, SD 14.16 years). All participants also had public Twitter profiles, lived in the United States, and gave permission to monitor their public Twitter profile. People with public Twitter profiles were recruited to explore patterns of information sharing and consumption related to e-cigarettes on Twitter, which will be reported in a forthcoming paper. The majority of e-cigarette users in the sample (245/278, 88.1%) were also current smokers. It is important to note that in this study e-cigarette users who were smokers were classified as e-cigarette users so that exposure to and sharing of e-cigarette tweets could be assessed for smokers who had not tried e-cigarettes, but may be using Twitter to learn about e-cigarettes. The study was approved by RTI International’s Institutional Review Board.

### Recruitment Method

Equal numbers of participants were intentionally recruited via Qualtrics’ panel aggregator (n=190) or promoted tweets (n=190).

#### Panel Recruitment

Qualtrics panel aggregator was used to recruit online panel participants for this study. The Qualtrics panel aggregator provides clients with access to members of a number of market research panels and uses digital fingerprinting technology and IP address checks to ensure that participants’ data are as valid and reliable as possible. Participants recruited via panel received an email from Qualtrics inviting them to participate in the study by clicking on a link to a screening questionnaire to assess eligibility. Participants recruited via the Qualtrics panel aggregator were targeted based on profiling attributes that are included in online panels that are used to guarantee that data about panel respondents are detailed and accurate. We used the following profiling attributes provided previously by participants to target participants for the survey: age, being a smoker, and being part of an online social network. Qualtrics did not have the capability to target e-cigarette users; thus, participants were not targeted based on their e-cigarette use (see Table 1 for targeting features). Panel recruitment was initiated with a soft launch that began 2 weeks before the launch of Twitter ads for recruitment and continued after the launch of Twitter ads.

#### Twitter Recruitment

**Figure 1:**
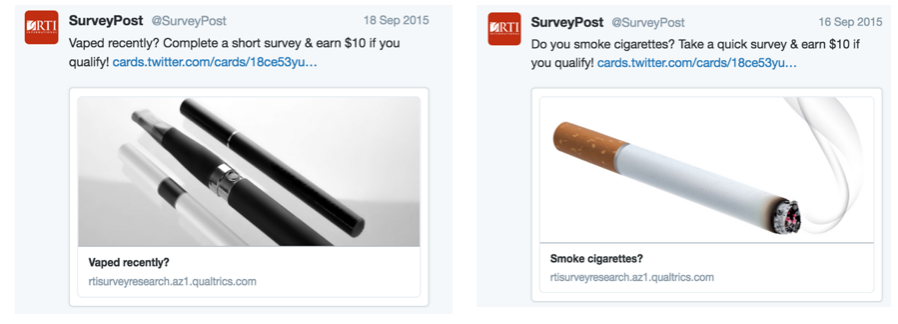
Twitter ad examples targeting e-cigarette smokers (left) and cigarette smokers (right).

Twitter ads targeted e-cigarette users and smokers using two separate campaigns for each user group. Each ad included a brief description (e-cigarette users: “Vaped recently? [or “Ever vape?” or “Ever use e-cigarettes?”] Complete a short survey & earn $10 if you qualify!”; smokers: “Smoked recently? [or “Do you smoke cigarettes?”] Take a quick survey & earn $10 if you qualify!”), an image (e-cigarette users: e-cigarettes; smokers: cigarettes), and a link to the screening questionnaire (see Twitter ad examples in Figure 1). Twitter ads were posted by the RTI Twitter handle SurveyPost (Twitter handle used for conducting survey research at RTI), which displayed the RTI logo. These ads showed up as promoted tweets in targeted users’ Twitter feeds.

Twitter ads provide a number of targeting capabilities for reaching a specific target audience. Targeting features used for ads included (1) age targeting ads to adults aged 18 or older, and (2) keyword and hashtag targeting for words and hashtagged words that Twitter users have tweeted or searched for on Twitter related to e-cigarettes and smoking (see Table 1 for targeting keywords).

**Table 1:** Recruitment targeting strategies by recruitment method.

Targeting feature	Panel	Twitter
Age	18 years or older	18 years or older
Smoker targeting	Smokers (smoking habit or smoke at least once per day)	Keywords/hashtags: (#)cigarettes, (#)smoking, (#)tobacco, (#)cigarette, smoker, (#)smokers, tobacco smoking, cig, cigs, ciggy
E-cigarette user targeting	No targeting available	Keywords/hashtags: e-cig, ejuice, eliquid, (#)vape, (#)vaping, (#)ecigs, (#)ecig, ecigarette, e-liquid, vaper, #vaping, #vape, #vapelyfe, vapes, #vapeislife, #vapeon, #vapelife, #vapeon
Social network use	Member of at least one online social network	Twitter users (ads appeared on Twitter)

### Dependent Variable

Recruitment method (Twitter, panel) for completed surveys was the dependent variable.

### Procedure

#### Panel Recruitment

Qualtrics sent email invitations to panel members who met the targeting recruitment criteria (aged 18 or older, part of an online social network, and/or smokers [to recruit smokers and/or e-cigarette users who may or may not be smokers]). The email invitation included a link to the screening questionnaire that participants then clicked and completed to assess their eligibility for participating in the survey. A feature was enabled in the survey that prevented any person with the same IP address from completing the survey more than once to prevent duplicate responses. As an added precaution to prevent duplicate responses, respondent email addresses were cross-checked for both exact email address matches and similar name matches (eg, jdoe@gmail.com, jdoe@yahoo.com, jdoe1@gmail.com) with a database of emails from participants who had already completed the survey (via Qualtrics panel or Twitter) to prevent participants who completed the survey multiple times from receiving multiple incentives and from being included in the final sample. Participants determined to be eligible based on their responses to screening questions related to age, having a public Twitter profile, and being e-cigarette users or smokers were presented with a brief consent form. Individuals who consented to participate in the study continued directly to the 20-minute Web survey where they answered questions about their demographic characteristics, tobacco product use (cigarettes, other tobacco products, e-cigarettes), cessation behaviors, e-cigarette-related perceptions, social media and Internet use, and exposure to/recall of e-cigarette content on social media. Participants who completed the survey were compensated with the standard Qualtrics panel incentive, which can be redeemed for rewards and had an estimated value of approximately US $1.50. This incentive structure and amount is standard procedure for what Qualtrics and other survey panels provide to participants as compensation for this length of survey.

#### Twitter Recruitment

Initial contact with potential participants occurred through Twitter ads (ie, promoted tweets) targeted at participants who were likely to be eligible using age and keyword targeting. Participants recruited via Twitter clicked on a promoted tweet in their Twitter feed and were then directed to a Web link for the screening questionnaire. The same feature was enabled in the survey that prevented any person with the same IP address from completing the survey more than once to prevent duplicate responses. The same email cross-checking procedure was used as a second precaution to prevent duplicate respondents from receiving multiple incentives and being included in the final sample. Participants then completed the same screening questionnaire as those recruited via panel, and eligible participants completed the same consent form and survey instrument as panel participants. Participants recruited via Twitter who completed the survey were compensated with a US $10 digital gift card incentive. Qualtrics incentives were the standard incentive provided for the length of survey administered and could not be altered to match incentives for Twitter participants.

### Predictor Variables

Independent variables were demographics (age, gender, education, race/ethnicity), e-cigarette use (past 30-day e-cigarette use, e-cigarette puffs per day, time to first e-cigarette), and social media use (eg, Twitter use frequency, using Twitter to give and receive e-cigarette advice, using Twitter to learn about e-cigarettes, posting or sharing information about e-cigarettes online).

### Statistical Analysis

Unpaired sample means tests were used to compare the percentage of people from Twitter versus the panel who completed each stage of the recruitment process. To determine which recruitment method was more efficient in recruiting participants, a series of Poisson regression analyses were conducted on the number of eligible participants who completed the full survey and provided public Twitter data and the recruitment method (Twitter vs panel) with days to survey completion (from the first day of data collection until the goal sample of 190 participants was reached for each recruitment method) for each recruitment method included as an offset variable [15]. Using the Poisson regression with days to completion as an offset variable allows for computation of recruitment efficiency as an incidence rate ratio (IRR) for time to completion. Recruitment method was the predictor in the model. Two Poisson models were conducted to compare the efficiency of the two recruitment methods. The first model included all survey completes from the 2-week soft launch of panel recruitment that occurred before the launch of Twitter ads and all completes thereafter. The second included only survey completes that occurred when both recruitment methods were active (excluding all survey completes from the 2-week panel soft launch).

A series of bivariate analyses were conducted to determine which variables differed between people recruited via Twitter and online panel. Variables related to recruitment source (*P*<.25) were included in multivariate logistic regression models [29]. Analyses were run in Stata version 13.1. Predictors included demographics, e-cigarette use variables (e-cigarette users only), and social media use variables.

## Results

The Twitter ad campaigns used to recruit e-cigarette users and smokers generated a total of 590,954 impressions (ie, individual exposures to an ad) with 395,035 and 195,919 impressions generated from the smoker and e-cigarette targeted ad campaigns, respectively. Ads resulted in 2691 total clicks, with 1718 clicks on smoker-targeted ads (0.43%, 1718 clicks/395,035 exposures) and 973 clicks on e-cigarette user-targeted ads (0.50%, 973 clicks/195,919 exposures). Total cost of ads was US $6848.25 (US $4206.23 for smoker-targeted ads and US $2642.02 for e-cigarette user-targeted ads). Qualtrics panel sent 152,221 email invitations to panel members who met the target recruitment criteria and 15,262 panel members clicked on the survey link, demonstrating that emails sent via panel resulted in a 10.00% survey click rate. Cost comparisons could not be made between the two recruitment methods because Qualtrics was used for both panel recruitment and for programming and managing the survey (completed by all participants) and they do not provide a cost breakdown that separates out these overlapping costs to determine the cost of panel recruitment in isolation.

### Recruitment Efficiency

**Figure 2:**
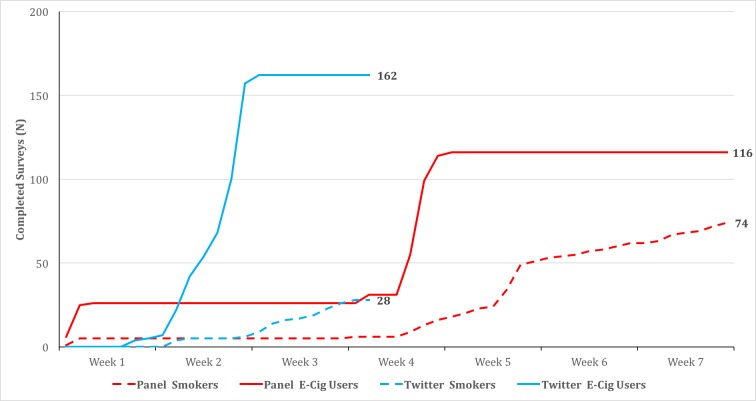
Timeline for completed surveys among e-cigarette users (n=190) and smokers (n=190) by recruitment method (Twitter or panel).

Although participants were recruited in equal numbers via panel and Twitter (n=190 each), results demonstrated that the IRR for time to completion was 1.30 times faster for Twitter participants than panel participants (*P*=.02) when including only survey completes that were received during active recruitment for both Twitter and panel, and 2.13 times faster (*P*<.001) when also including survey completes from the 2-week panel soft launch that occurred before the Twitter ads launched. Figure 2 illustrates the trajectory of survey completions for each recruitment method for e-cigarette users and smokers.

### Eligibility and Survey Completion

A larger percentage of people recruited via panel completed the screener than people recruited via Twitter (*P*=.02) (see Table 2 for n’s and percentages). Of the participants who completed the screening questionnaire, the proportion of participants in the eligible age range did not differ significantly based on recruitment method, although a larger proportion of participants recruited via Twitter were eligible based on e-cigarette use or smoking behavior than those recruited via panel (*P*<.001). Compared with participants recruited via Twitter, a larger percentage of participants recruited via panel (1) provided their Twitter handle and consented to share their public Twitter data (*P*=.002), and (2) consented to complete the online survey (*P*<.001). Finally, compared to those recruited via panel, a larger proportion of participants recruited via Twitter (1) provided a public Twitter handle that we were able to use to extract their Twitter data (*P*<.001), and (2) completed the baseline survey (*P*<.001). Taken together, these findings show that Twitter recruitment resulted in a higher proportion of useable data and completed surveys from Twitter.

**Table 2:** Eligibility, baseline, and follow-up completion by recruitment method.

Stage of completion	Total		Panel		Twitter		*P* value
	n (%)^a^	95% CI	n (%)^a^	95% CI	n (%)^a^	95% CI	
Completed screener	2155 (51.9)	50.4-53.3	1587 (52.9)	51.2-54.6	568 (48.8)	45.9-51.8	.02
Eligible: age	2154 (100)	99.9-100	1586 (99.9)	99.8-100	568 (100)	—	.32
Eligible: e-cigarette use/smoker	2024 (94.0)	93.0-95.0	1474 (92.9)	91.7-94.2	550 (96.8)	95.4-98.3	<.001
Consented to share Twitter data	2014 (99.5)	99.2-99.8	1474 (100)	—	540 (98.2)	97.1-99.3	.002
Consented to survey	1926 (95.6)	94.7-96.5	1452 (98.5)	97.9-99.1	474 (87.8)	85.0-90.5	<.001
Completed survey	919 (47.7)	45.5-49.9	604 (41.6)	39.1-44.1	315 (66.5)	62.2-70.7	<.001
Public Twitter handle	380 (41.3)	38.2-44.5	190 (31.5)	27.7-35.2	190 (60.3)	54.9-65.7	<.001

^a^ Denominator for each column percentage is the numerator from the preceding row, with the exception of “completed screener” which uses the total number of screeners (complete and incomplete) as the denominator (total: N=4479; panel: n=3369; Twitter: n=1110).

### Sample Characteristics

Characteristics of the participants are presented in Table 3.

Overall, the majority of the sample was white, non-Hispanic (255/380, 67.1% vs 77.1% in 2015 US census), with a larger percentage of white, non-Hispanic smokers (76/102, 74.5% ) compared with e-cigarette users (179/278, 64.4%). Among the 16.6% (63/380) of Hispanics (vs 17.6% in 2015 US census) in the sample, a larger percentage were e-cigarette users (55/278, 19.8%) than were smokers (8/102, 7.8%). Among the 5.8% (22/380) of black, non-Hispanic participants (vs 12.6% in 2015 US census), a larger percentage were smokers (8/102, 7.8%) than e-cigarette users (14/278, 5.0%). Overall, the sample was almost evenly split by gender (52.4%, 199/380 male vs 49.2% in 2015 US census), but a larger percentage of e-cigarette users were male (160/278, 57.6%) and a larger percentage of smokers were female (63/102, 61.8%). A smaller percentage of participants had a college degree or higher (127/380, 33.4% vs 29.3% in the 2015 US census); this was true of both e-cigarette users (95/278, 34.2%) and smokers (32/102, 31.4%).

The majority of e-cigarette users reported using their first e-cigarette 30 minutes or less after waking (118/189, 62.4%). On average, e-cigarette users reported using e-cigarettes on a mean 13.99 (SD 11.99) days of the past 30 days and taking a mean 54.68 (SD 139.66) puffs per day on their e-cigarette.

On average, participants were most likely to report using Twitter daily or several times per day (mean 6.68, SD 1.94), with smokers reporting slightly higher mean Twitter use rates on average (mean 7.13, SD 1.70) than e-cigarette users (mean 6.51, SD 2.00), although this difference was not statistically signitifcant. Less than half of the sample reported using Twitter to give and receive advice about e-cigarettes (161/380, 42.4%) and to learn about e-cigarettes (171/380, 45.0%); these percentages were higher among e-cigarette users (advice: 135/278, 48.6%; learn: 142/278, 51.1%) than smokers (advice: 26/102, 25.5%; learn: 29/102, 28.4%; *P*<.001). More than one-third of participants reported that they post and share information about e-cigarettes online (146/380, 38.4%), and this number was higher among e-cigarette users (129/278, 46.4%) than smokers (17/102, 16.7%) (*P*<.001).

### Bivariate Analyses

As predicted, smokers were more likely to be recruited via panel (74/102, 72.6%) than via Twitter (28/102, 27.5%, *P*<.001). Also in line with expectations, e-cigarette users were more likely to be recruited via Twitter (162/278, 58.3%) than panel (116/278, 41.7%, *P*=.01).

Overall, e-cigarette users and smokers tended to be older and male. More specifically, bivariate analyses showed that adult e-cigarette users and smokers aged 25 to 34 years (e-cigarette users: OR 0.11, 95% CI 0.04-0.29, *P*<.001; smokers: OR 0.10, 95% CI 0.02-0.61, *P*=.01), 35 to 44 years (e-cigarette users: OR 0.09, 95% CI 0.03-0.27, *P*<.001; smokers: OR .01, 95% CI 0.00-0.18, *P*=.001), 45 to 54 years (e-cigarette users: OR 0.05, 95% CI 0.02-0.17, *P*<.001; smokers: OR .08, 95% CI 0.01-0.51, *P*<.01), and 55 years or older (e-cigarette users: OR 0.06, 95% CI 0.02-0.21, *P*<.001; smokers: OR 0.08, 95% CI 0.01-0.48, *P*=.01) were less likely to be recruited via Twitter (than panel) compared with young adult e-cigarette users aged 18 to 24 years. Male e-cigarette users (OR 1.92, 95% CI 1.18-3.12, *P*=.01) and smokers (OR 2.40, 95% CI 0.99-5.84, *P*=.05) were more likely than women to be recruited by Twitter than panel. E-cigarette users who reported a college education or greater were less likely to be recruited via Twitter than panel (OR 0.34, 95% CI 0.20-0.57, *P*<.001) compared to those with less education. E-cigarette users who reported using e-cigarettes on more days of the past 30 (OR 1.05, 95% CI 1.03-1.08, *P*<.001), who took more puffs on their e-cigarette per day (OR 1.08, 95% CI 1.05-1.11, *P*<.001), and who used e-cigarettes more than 30 minutes after waking (OR 2.08, 95% CI 1.11-3.88, *P*=.02) were more likely to be recruited via Twitter than panel.

As expected, e-cigarette users and smokers who reported using Twitter more frequently were more likely to be recruited via Twitter than panel, such that with each one-unit increase in Twitter usage, e-cigarette users were 1.93 times as likely to be recruited via Twitter than panel (95% CI 1.53-2.42, *P*<.001), and smokers were 3.69 times as likely to be recruited via Twitter than panel (95% CI 1.72-7.92, *P*=.001). E-cigarette users who reported using Twitter to give or receive advice about e-cigarettes were less likely to be recruited via Twitter than panel (OR 0.47, 95% CI 0.28-0.76, *P*=.002).

**Table 3:** Sample characteristics.

Characteristic			Total sample (N=380)	E-Cigarette users (n=278, 73.2%)	Smokers (n=102, 26.8%)	*P* value
**Demographics**						
	Age (years), mean (SD)		36.55 (13.61)	34.26 (14.16)	42.80 (14.16)	<.001
	**Gender, n (%)**					
		Female	181 (47.6)	118 (42.5)	63 (61.8)	.001
		Male	199 (52.4)	160 (57.6)	39 (38.2)	
	**Education, n (%)**					
		Less than college	253 (66.6)	183 (65.8)	70 (68.6)	.61
		College plus	127 (33.4)	95 (34.2)	32 (31.4)	
	**Race/ethnicity, n (%)**					
		White, non-Hispanic	255 (67.1)	179 (64.4)	76 (74.5)	.05
		Black, non-Hispanic	22 (5.8)	14 (5.0)	8 (7.8)	.35
		Hispanic	63 (16.6)	55 (19.8)	8 (7.8)	.001
		Other/multiple races	40 (10.5)	30 (10.8)	10 (9.8)	.78
**E-Cigarette use (e-cigarette users only)**							
	Past 30-day e-cigarette use (n=205), mean (SD)		—	13.99 (11.99)	—	
	E-cigarette puffs per day (n=116), mean (SD)		—	54.68 (139.66)	—	
	**Time to first e-cigarette**							
		≤30 minutes	—	118 (62.4)	—	
		>30 minutes	—	71 (37.6)	—	
**Cigarette use**						
	Smoked 100 cigarettes in lifetime, n (%)		371 (97.6)	269 (96.8)	102 (100)	<.001
	**Do you now smoke cigarettes...?, n (%)**						
		Every day	256 (67.4)	168 (60.4)	88 (86.3)	<.001
		Some days	80 (21.1)	66 (23.7)	14 (13.7)	.02
		Rarely	12 (3.2)	12 (4.3)	0 (0)	.001
		Not at all	32 (8.4)	32 (11.5)	0 (0)	<.001
**Social media use**							
	Twitter usage (1=never, 8=several times a day), mean (SD)		6.68 (1.94)	6.51 (2.00)	7.13 (1.70)	.37
	Use Twitter to give/receive e-cigarette advice, n (%)		161 (42.4)	135 (48.6)	26 (25.5)	<.001
	Use Twitter to learn about e-cigarettes, n (%)		171 (45.0)	142 (51.1)	29 (28.4)	<.001
	Post/share information about e-cigarettes online, n (%)		146 (38.4)	129 (46.4)	17 (16.7)	<.001

### Logistic Regression Analyses

Logistic regression models were used to compare the demographic characteristics, e-cigarette use, and social media use between people recruited via Twitter and panel (Table 4). Variables found to be related to recruitment source in bivariate analyses were included in the multivariate models (*P*<.25) [24]. As hypothesized, e-cigarette users aged 25 to 34 years (OR 0.01, 95% CI 0.00-0.60, *P*=.03) and 35 to 44 years (OR 0.01, 95% CI 0.00-0.51, *P*=.02) were less likely to be recruited via Twitter than panel compared with e-cigarette users aged 18 to 24 years. This difference did not emerge when comparing the 18 to 24 group to older adults (older than 45 years). Similarly, in line with our hypotheses, smokers aged 35 to 44 years were less likely than those aged 18 to 24 years to be recruited via Twitter than panel (OR 0.03, 95% CI 0.00-0.49, *P*=.01). E-cigarette users who reported a greater number of puffs on their e-cigarette per day were more likely to be recruited via Twitter than panel compared with e-cigarette users who reported fewer puffs per day (OR 1.12, 95% CI 1.05-1.20, *P*=.001). In addition, with each one-unit increase in Twitter usage, e-cigarette users were 9.55 times (95% CI 2.28-40.07, *P*=.002) and smokers were 4.91 times (95% CI 1.90-12.74, *P*=.001) as likely to be recruited via Twitter than panel.

**Table 4:** Multivariate logistic regressions of e-cigarette users and smokers recruited via Twitter (versus panel).^a^

Variable			E-Cigarette users (n=278)		Smokers (n=102)	
			AOR (95% CI)	*P* value	AOR (95% CI)	*P* value
**Demographics**						
	**Age**					
		18-24 years	REF		REF	
		25-34 years	0.01 (0.00-0.60)	.03	0.23 (0.03-1.65)	.15
		35-44 years	0.01 (0.00-0.51)	.02	0.03 (0.00-0.49)	.01
		45-54 years	0.02 (0.00-1.98)	.09	0.47 (0.06-3.88)	.48
		≥55 years	0.02 (0.15-8.13)	.08	0.34 (0.04-2.73)	.31
	**Gender**					
		Female	REF		REF	
		Male	1.10 (0.02-7.41)	.92	3.25 (0.84-12.55)	.09
	**Race/Ethnicity**					
		White, non-Hispanic	REF		REF	
		Black, non-Hispanic	1.00 (—)	—	3.24 (0.37-28.19)	.29
		Hispanic	0.41 (0.02-7.41)	.54	1.01 (0.07-14.71)	.99
		Other/multiple races	9.04 (0.30-274.33)	.21	3.00 (0.48-18.58)	.24
	**Education**					
		Less than college	REF		REF	
		College plus	0.17 (0.03-1.03)	.05	2.01 (0.58-6.96)	.27
**E-Cigarette use (e-cigarette users only)**						
	Past 30-day e-cigarette use		1.02 (0.93-1.12)	.67	—	—
	E-cigarette puffs per day		1.12 (1.05-1.20)	.001	—	—
	**Time to first e-cigarette**						
		≤30 minutes	REF		—	—
		>30 minutes	1.48 (0.20-10.72)	.70	—	—
**Social media use**						
	Twitter usage		9.55 (2.28-40.07)	.002	4.91 (1.90-12.74)	.001
	**Use Twitter to give/receive e-cigarette advice**						
		No	REF		—	—
		Yes	0.28 (0.03-2.40)	.25	—	—

^a^ Predictors include variables related to recruitment methods in univariate analyses (*P*<.25).

For both e-cigarette users and smokers, those aged 18 to 24 years and individuals who were heavier users of Twitter were more likely to be recruited via Twitter than adults aged between 35 and 44 years. E-cigarette users aged between 18 and 24 years were also more likely than those aged between 25 and 35 years to be recruited via Twitter, but the same was not true of smokers.

## Discussion

### Principal Results

E-cigarette users and smokers were recruited more quickly via Twitter than online panel. A larger percentage of people recruited via Twitter were eligible to participate in the study based on their e-cigarette use or smoking behavior compared with participants recruited via panel, suggesting that Twitter recruitment provided a more direct way (ie, requiring that fewer people need to be screened to reach eligible participants) to reach the target populations. Participants recruited via panel were more likely to consent to participate in the survey than those recruited via Twitter, which makes sense because panel members are already experienced with completing online surveys for incentives. In contrast, a larger percentage of participants recruited via Twitter completed the survey and provided public Twitter handles (which was an eligibility requirement for accessing their Twitter data) compared to those recruited via panel.

Consistent with our predictions, as well as research suggesting that smoking rates are highest among adults aged between 25 and 44 years [23] and that panel use is higher among older adults [22], our findings demonstrated that smokers were more likely to be recruited via panel. Similarly, in line with research showing e-cigarette and Twitter use rates to be highest among young adults [27,28], findings show that e-cigarette users were more likely to be recruited via Twitter.

Twitter and online panel recruitment methods provided access to different subgroups of e-cigarette users and smokers. Consistent with our predictions and research showing that Twitter is more popular among younger adults [27], Twitter ads recruited e-cigarette users and smokers who were younger and were heavier users of Twitter than people recruited via panel. Twitter also recruited e-cigarette users who reported taking more e-cigarette puffs per day than people recruited via panel. Recruiting participant populations via Twitter along with online panel offered access to a more diverse population than using a single recruitment method.

Overall, findings from this study suggest that recruiting participants directly from Twitter provided the most effective means of accessing people who would both complete a survey and a provide public Twitter handles for extracting Twitter data. In addition, participants recruited via Twitter reported being heavier users of Twitter, suggesting that recruiting participants in this way provides access to a population for whom questions about e-cigarette information exposure and sharing on Twitter are most relevant.

Twitter provides highly specific targeting features that allow users to be targeted based on demographics, interests, and other characteristics, which in the case of this research included age and use of keywords related to e-cigarettes and smoking. These features make Twitter a more efficient resource for reaching the target population than an online panel because panel participants could only be targeted based on age, membership in any online social network, and smoking behavior (and could not be targeted based on e-cigarette use).

### Comparison With Prior Work

This study expands the literature on using social media to recruit hard-to-reach populations in several ways. First, this study demonstrates the efficacy of using Twitter for participant recruitment, which has been shown in few published research studies to date [25]. Second, to our knowledge, this is the first study to collect both users’ self-reported survey data and their social media data in combination to provide a more holistic picture of how participants who provide data from this combination of sources differ based on demographic and other characteristics, and how it may influence perceptions and behavior. These data will be used in a separate, forthcoming paper to illuminate how information about an emerging product is disseminated on social media. Third, this research expands the literature on comparing social media recruitment methods to traditional recruitment methods by demonstrating important differences in recruitment effectiveness and efficiency, and demographic and other characteristics of participants recruited via Twitter compared with an online panel.

### Limitations

Although this study provides important insights into the usefulness of online panels and Twitter for recruiting hard-to-reach participant populations, this research has several limitations. First, both samples recruited for this study are not representative of the US population of e-cigarette users and smokers, and findings may not generalize to a national sample of e-cigarette users and smokers. Second, only one online panel provider was compared to Twitter recruitment and, thus, findings may not generalize to recruitment efforts using other online panels. Third, people could have been exposed to both Twitter and panel recruitment materials because recruitment efforts for the study were conducted simultaneously. Fourth, panel and Twitter incentives provided to participants were not equivalent (panel participants received the standard incentive of panel points and Twitter participants received a US $10 digital gift card) suggesting the possibility that the findings reported here may be driven by differences in incentives received by participants between the two recruitment methods. Fifth, cost comparisons could not be made between the two recruitment methods to determine whether one method is more cost efficient for participant recruitment. A final limitation of this research, and any research conducted using social media to recruit participants [15], is that the algorithms used for ad placement on social media are based on private user data and are constantly changing, making it difficult for researchers to determine which participant characteristics are most important for targeting advertisements to a desired participant population.

### Conclusions

Our findings demonstrate that Twitter ads were more efficient than an online panel in recruiting e-cigarette users and smokers with a substantially larger number of eligible participants completing surveys and other eligibility requirements. In addition, Twitter and online panels provide access to different subgroups of these hard-to-reach populations. Twitter provided access to younger adults, who were heavier users of Twitter and e-cigarettes (e-cigarette users only). Recruiting participants via social media along with online panel offered access to a broader population from which to understand e-cigarette use than would one of the two recruitment sources alone.

## Abbreviations

IRR: incidence rate ratio
